# Evaluating researcher and stakeholder perspectives on socially responsible research and innovation policies and practices in marine and maritime research performing organisations

**DOI:** 10.12688/openreseurope.14325.2

**Published:** 2023-07-20

**Authors:** Eric A. Jensen, Lars Lorenz

**Affiliations:** 1 Thriving Natural Capital Challenge Centre, SRUC, Edinburgh, Scotland, UK; 2 Institute for Methods Innovation, Dublin, Ireland

**Keywords:** RRI, socially responsible science, researchers, scientists, indicators, evaluation

## Abstract

The European Commission-funded GRRIP (Grounding RRI Practices) project aims to embed sustainable Responsible Research and Innovation (RRI) practices in five research performing organisations (RPOs), focusing on the marine and maritime sector. The project’s goal is to achieve institutional and cultural change through a cycle of evaluation, evidence-based interventions and further evaluation. For this purpose, a set of three surveys were designed and implemented in the first part of the project (2020) to establish a baseline measurement of RRI-related practices within the project partner institutions and their stakeholders. Each survey was specifically designed to target a relevant category of people for each of the five RPOs implementing RRI actions. These five institutions are research departments and centres linked to the marine and maritime sector in Ireland, Spain, Portugal, France and the UK. This paper presents the design of these survey-based evaluation instruments and the linked datasets generated by their implementation.

## Plain language summary

There has been a longstanding movement in the global research community to make research and innovation more useful to society. These efforts have been linked to a wide range of approaches to make research practices more socially responsible, that is, more inclusive, transparent and publicly accessible. This paper focuses on a particular aspect of such initiatives, namely, how to measure progress towards more socially responsible research practices and policies at the level of research institutions. Here, specific approaches to doing this with survey methods are described, alongside the data from an effort to implement these surveys in five research organisations across Europe.

## Introduction

The European Commission-funded GRRIP (Grounding RRI Practices) project aims to embed sustainable Responsible Research and Innovation (RRI) practices in five research performing organisations (RPOs), focusing on the marine and maritime sector. The project’s goal is to achieve institutional and cultural change through a cycle of evaluation, evidence-based interventions and further evaluation. For this purpose, a set of three surveys were designed and implemented in the first part of the project to evaluate current RRI practices within the project partner institutions and their stakeholders. Each survey was specifically designed to target a relevant category of people for each of the five RPOs implementing RRI actions. These five institutions are research departments and centres linked to the marine and maritime sector in Ireland, Spain, Portugal, France and the UK, each of which was a partner on the GRRIP project:

- The Centre for Marine and Renewable Energy (MaREI), University College Cork, Ireland
- Plataforma Oceánica de Canarias (Plocan), Gran Canaria
- WavEC Offshore Renewables, Lisbon, Portugal
- Institut Universitaire Mer Littoral U.Nantes (IUML), France
- Swansea University, Wales

## Materials and methods

### Research instruments

***RPO survey.*** The RPO survey was designed to evaluate the presence and availability of RRI-related policy and guidance within each of the five marine and maritime research organisations. Respondents were asked to indicate whether policy or guidance covering a specific RRI dimension was available within their institution and also provide the full documents or text passages of relevant policies accordingly. The RRI-related topics covered in this survey were:

- Gender Equality
- Open Access Publications
- Public Engagement
- Research Ethics and Integrity
- Science Education and Outreach

In addition to the documentation of reported RRI-related policies and guidance, the survey also asked whether the respondent’s organisation had any dedicated staff, meetings, training or other organisational structures in place to monitor and enable RRI practices.

***Researcher and stakeholder surveys.*** Both designed to deliver RRI indicators ( Jensen & Lister, 2015) for impact evaluation purposes (e.g., Jensen, 2015; Jensen, 2020), the researcher and stakeholder surveys (described below) were aligned to work in tandem and are therefore similar in several ways. However, the researcher survey was targeted towards people who work in research performing organisations in the marine and maritime sector, while the stakeholder survey was aimed at those who have links or collaborations with the five RPOs in the project to find out whether RRI was being implemented in a way that was recognised by stakeholders.

The content of the two surveys focused on the policies and practices employed by each of the five marine and maritime organisations involved in the project. It was informed by recent research and conceptualization of responsible research. Respondents were first asked to provide some personal information about themselves and their career, such as employment status, experience, and scientific field of work. Furthermore, they were asked about how they incorporate RRI practices in their own work and how much time they dedicate to engage in both research and research-related work (e.g., communication, management or administration). The survey then proceeded to gather information about respondents' views on the RPOs’ implementation of a range of different RRI dimensions, such as workforce diversity, gender equality, inclusion of ethnic minorities, alignment with societal needs and ethics.

### Data collection

Respondents were recruited differently for each survey. The RPO survey was completed by designated representatives of the five marine and maritime RPOs in the project implementing RRI actions. The researcher survey was completed by people who worked at these same RPOs, while the stakeholder survey was sent to people linked to the RPOs but working in a range of sectors. A description of the project and the reasons for participation were included to enable respondents to self-assess their relevance to the survey request and decide whether to complete the survey. These participants were recruited by the project representatives at each of these sites through department or institution wide requests for participation. These requests were distributed by those involved in the project (which focuses on institution and department level policies and practices for socially responsible research in the marine and maritime context). No inclusion/exclusion criteria for the sample were applied beyond this. Specific information about the type of researchers or stakeholders responding were captured within the survey to be considered in the analysis. Survey data were collected March - June 2020.

The dataset collected using the instruments described above was cleansed according to established social research good practice (e.g., Jensen & Laurie, 2016; Smith & Jensen, 2016). As a first step, all data was completely anonymised where personally identifiable information was provided by respondents. Further, all insufficiently completed responses were removed, such as respondents who discontinued the survey after a few questions. It is available in both SPSS and CSV formats to maximise compatibility across different devices and research tools ( Lorenz & Jensen, 2021a; Lorenz & Jensen, 2021b; Lorenz & Jensen, 2021c). The survey data collection operated with a focus on evaluation. Therefore, the goal was to gain a broad base of participation, as much as was feasible during the available timeframe. There were challenges in gathering the external stakeholder sample, with relatively low participation rates. This suggests that efforts to gain participation in situations where stakeholders are not already heavily engaged by research organisations require greater investment in participant compensation and an associated recruitment campaign (in this case, no incentives were offered.

Due to the sampling approach, the datasets themselves cannot be taken as fully representative samples of their institutions, and certainly not of European research institutions more generally. Rather, these data offer an initial exploration of the status of these particular institutions on a number of different metrics at the outset of a culture change process aimed at bolstering the level of breadth of socially responsible research and innovation policies and practices at these locations. These data were used as an ‘audit’ tool to establish where there were gaps needing improvement in the five research performing organisations. In addition, the surveys provided baseline data that allowed for impact evaluation of the 4-year project’s efforts to improve policies and practices.

## Ethics policies and consent

The data were collected under the auspices of ethics approval by the relevant University College Cork institutional review board, the Social Research Ethics Committee (application date: 14 August 2019) for the GRRIP project. Written informed consent for publication of the participants’ details and/or their images was obtained from the participants in all cases, in line with the General Data Protection Regulation.

## Data Availability

Zenodo: GRRIP WP5 - RPO Survey. https://doi.org/10.5281/zenodo.5708901 ( Lorenz & Jensen, 2021a). This project contains the following underlying data: GRRIP_RPO_survey_data_documentation.pdf Zenodo: GRRIP WP5 - Researcher Survey. https://doi.org/10.5281/zenodo.5708878 ( Lorenz & Jensen, 2021b). This project contains the following underlying data: GRRIP_researcher_survey_data_CSV.csv GRRIP_researcher_survey_data_documentation.pdf GRRIP_researcher_survey_data_SPSS.sav GRRIP_researcher_survey_structure.csv Zenodo: GRRIP WP5 - Stakeholder Survey. https://doi.org/10.5281/zenodo.5708883 ( Lorenz & Jensen, 2021c). This project contains the following underlying data: GRRIP_stakeholder_survey_data_CSV.csv GRRIP_stakeholder_survey_data_documentation.pdf GRRIP_stakeholder_survey_data_SPSS.sav GRRIP_stakeholder_survey_structure.csv Data are available under the terms of the Creative Commons Attribution 4.0 International license (CC-BY 4.0). GRRIP WP5 - RPO Survey full datasets have not been made publicly available because the project deemed the datasets generated from this survey to be confidential.

## References

[ref-1] Jensen E : Highlighting the value of impact evaluation: Enhancing informal science learning and public engagement theory and practice. *JCOM J Sci Commun.* 2015;14(3):Y05. 10.22323/2.14030405

[ref-2] Jensen EA : How should socially responsible science be measured? (eLetter). *Science.* 2020;369(6499). 10.1126/science.abb3415

[ref-3] Jensen E Laurie C : Doing real research: A practical guide to social research. SAGE: London,2016. Reference Source

[ref-4] Jensen EA Lister TP : Evaluating indicator-based methods of ‘measuring long-term impacts of a science center on its community’. *J Res Sci Teach.* 2015;53(1):60–64. 10.1002/tea.21297

[ref-5] Lorenz L Jensen E : GRRIP WP5 - RPO Survey (1.0) [Data set]. *Zenodo.* 2021a. 10.5281/zenodo.5708901

[ref-6] Lorenz L Jensen E : GRRIP WP5 - Researcher Survey (1.0) [Data set]. *Zenodo.* 2021b. 10.5281/zenodo.5708878

[ref-7] Lorenz L Jensen E : GRRIP WP5 - Stakeholder Survey (1.0) [Data set]. *Zenodo.* 2021c. 10.5281/zenodo.5708883

[ref-8] Smith BK Jensen EA : Critical review of the United Kingdom’s “gold standard” survey of public attitudes to science. *Public Underst Sci.* 2016;25(2):154–170. 10.1177/0963662515623248 26783249

