# Supplementary material for: Evaluating researcher and stakeholder perspectives on socially responsible research and innovation policies and practices in marine and maritime research performing organisations
Source: Open Res Eur. 2023 Jul 20;1:150. Originally published 2021 Dec 14. [Version 2] doi: 10.12688/openreseurope.14325.2 (PMC11425032; doi:10.12688/openreseurope.14325.2)
Supplement: Peer review history

# Reviewer report: Reviewer response for version 2

**DOI:** 10.21956/openreseurope.17318.r44304

**Published:** 2024-09-27

**Authors:** Bernard D. Naughton

**Affiliations:** 1 The University of Dublin, Trinity College, Dublin, Ireland; 2 Kings College London,, London, England, UK

This data note provides a good overview of a broad preliminary survey to evaluate the extent of RRI practices among five research performing organisations.

 What is unclear to me  is to what extent the participants understood the concept of RRI, and if it was explained to them clearly as part of the survey?

 According to the RI  literature the main dimensions of RRI are anticipatory governance, inclusivity, reflexivity, responsiveness and responsible impact.

 Stilgoe, J., Owen, R., Macnaghten, P., 2013 (Ref -1). Developing a framework for responsible innovation. Research Policy 42, 1568–1580. https://doi.org/10.1016/j.respol.2013.05.008

 Naughton, B., Dopson, S., Iakovleva, T., 2023 (Ref-2). Responsible impact and the reinforcement of responsible innovation in the public sector ecosystem: cases of digital health innovation. Journal of Responsible Innovation 10, 2211870. https://doi.org/10.1080/23299460.2023.2211870

 I agree that, Gender Equality, Open Access Publications, Public Engagement, Research Ethics and Integrity, Science Education and Outreach can form part of  RRI but I would like to see a clearer link between these topics and the established RRI dimensions. I would suggest the authors include some text regarding what RRI is and to spell out which RRI dimensions the study topics are connected to.

Are sufficient details of methods and materials provided to allow replication by others?

Yes

Is the rationale for creating the dataset(s) clearly described?

Yes

Are the datasets clearly presented in a useable and accessible format?

Yes

Are the protocols appropriate and is the work technically sound?

Partly

Reviewer Expertise:

Responsible Innovation, Innovation Stigma, Pharmaceutical Regulatory Affairs

I confirm that I have read this submission and believe that I have an appropriate level of expertise to confirm that it is of an acceptable scientific standard.

## References

[1] : Developing a framework for responsible innovation. *Research Policy* .2013;42(9) : 10.1016/j.respol.2013.05.008 1568-1580 10.1016/j.respol.2013.05.008

[2] : Responsible impact and the reinforcement of responsible innovation in the public sector ecosystem: cases of digital health innovation. *Journal of Responsible Innovation* .2023;10(1) : 10.1080/23299460.2023.2211870 10.1080/23299460.2023.2211870

# Reviewer report: Reviewer response for version 2

**DOI:** 10.21956/openreseurope.17318.r44302

**Published:** 2024-09-25

**Authors:** Sergio Urueña Lopez

**Affiliations:** 1 Section of Philosophy, University of Twente, Enschede, Overijssel, The Netherlands; 2 Department of Philosophy, University of the Basque Country UPV/EHU, Donostia-San Sebastian, Spain

The Data Note outlines the work conducted through interviews aimed at establishing a baseline measurement of the implementation of Responsible Research and Innovation (RRI) practices and measures within five research-performing organizations (RPOs). The Data Note is well written and could be of great interest to various actors actively engaged with RRI or aiming to engage with it in the future.

 Below, I offer several constructive comments that I hope will help enhance the clarity and presentation of your work:

- There appears to be a discrepancy between the central focus of the Data Note as stated in the "Plain Language Summary" and the focus presented in the rest of the article. While the summary suggests that the surveys aim to measure *progress* toward RRI practices, the work instead provides an instrument designed to assess *the current state of RRI implementation* within RPOs at a specific point in time (i.e., a one-time ‘audit tool’). I recommend revising the "Plain Language Summary" to more accurately reflect the scope of the Data Note. While the survey instrument could potentially be used at different time points (i.e., through longitudinal surveys), enabling comparative analysis to assess progress in RRI implementation, this does not seem to be the primary focus as currently presented. If the authors plan to extend the evaluation to multiple time points and perform comparative analyses to track RRI implementation progress, it may be useful to include a brief note at the end of the article outlining these future possibilities and/or intentions (e.g., prior to the ‘Ethics Policies and Consent’ section).
- The introduction could benefit from a brief clarification of how “Responsible Research and Innovation” was understood during the development of the surveys. Over the past decade, the literature and debate on RRI have been extensive, with varying interpretations and approaches to its operationalization. The question of how RRI should be defined and understood remains open. Offering a foundational explanation of how RRI was conceptualized in the context of these surveys (e.g., based on the Horizon 2020 framework, including the “six pillars”) would help readers understand why themes such as “Gender Equality,” “Open Access Publications,” “Public Engagement,” “Research Ethics and Integrity,” and “Science Education and Outreach” were chosen as key focus areas in the surveys.
- Building on the previous point, if the authors have based their work on the six-pillar RRI framework, it would be helpful to clarify where the pillar of “Governance” fits within the survey. Was it incorporated into one of the five dimensions addressed, or was it intentionally excluded? If excluded, it would be valuable to provide the rationale for this decision.
- In the “Research and Instruments” section, the description of the interviews could be enriched with additional methodological detail. I recommend the authors elaborate briefly on the nature of the interviews conducted. For instance, it would be useful to clarify whether the surveys targeting RPOs, researchers, and stakeholders were: (a) cross-sectional or longitudinal (regarding the timing of data collection); (b) structured, semi-structured, or unstructured (regarding the organization of the questions); (c) administered online, by telephone, in person, or by post (regarding the mode of administration); and (d) self-administered or interviewer-administered. This additional detail would provide readers with a more comprehensive and nuanced understanding of the methodology used.

I look forward to seeing the final version and hope that my comments contribute positively to the refinement of this interesting work.

Are sufficient details of methods and materials provided to allow replication by others?

Yes

Is the rationale for creating the dataset(s) clearly described?

Partly

Are the datasets clearly presented in a useable and accessible format?

Partly

Are the protocols appropriate and is the work technically sound?

Partly

Reviewer Expertise:

Responsible innovation; Responsible Research and Innovation; Anticipation; Science and Technology Studies; Philosophy.

I confirm that I have read this submission and believe that I have an appropriate level of expertise to confirm that it is of an acceptable scientific standard, however I have significant reservations, as outlined above.

# Reviewer report: Reviewer response for version 2

**DOI:** 10.21956/openreseurope.17318.r33845

**Published:** 2023-07-31

**Authors:** Ross S. Bailie

**Affiliations:** 1 University Centre for Rural Health, University of Sydney, Lismore, NSW, Australia

The authors’ revisions look fine.

Are sufficient details of methods and materials provided to allow replication by others?

Partly

Is the rationale for creating the dataset(s) clearly described?

Yes

Are the datasets clearly presented in a useable and accessible format?

No

Are the protocols appropriate and is the work technically sound?

Yes

Reviewer Expertise:

Health services and systems research

I confirm that I have read this submission and believe that I have an appropriate level of expertise to confirm that it is of an acceptable scientific standard.

# Reviewer report: Reviewer response for version 1

**DOI:** 10.21956/openreseurope.15456.r28813

**Published:** 2022-04-06

**Authors:** Mercedes Ruiz-Lozano

**Affiliations:** 1 Department of Financial Economics and Accounting, Universidad Loyola Andalucía, Sevilla, Spain

This data note presents research that is very appropriate since the research centers must respond to the needs of society and also create value for it, but this can only be achieved by disseminating the results so that all stakeholders, involved or affected, can make decisions. Likewise, the centers that finance their research with public funds must be an example in the implementation of inclusion policies and ethical actions. There are some aspects that could be improved:

- The title of the research must mention the type of organizations analyzed.
- The objective is identified, but the reason for that objective is not clearly described. It is not described how the variables that are analyzed can respond to these objectives. With the surveys, guidelines for action will be identified. What will be the next steps?
- Organizations are clearly identified, but why these organizations have been chosen is not made clear. Is homogeneity sought in order to identify best practices or is diversity sought to identify possible patterns of action? It is not identified if the sample is representative and homogeneous by country.
- Science Education and Outreach. This topic is not clearly identified in the survey. What is it referring to? How has it been analyzed?
- The researchers indicate that the survey also asked whether the respondent’s organisation had any dedicated staff, meetings, training or other organisational structures in place to monitor and enable RRI practices. But these issues are not easily identified in the survey.
- The data is provided, but is not in a usable format because the data is accumulated in a single column. They should appear separated by rows and columns.

Thanks for sharing the research.

Are sufficient details of methods and materials provided to allow replication by others?

Yes

Is the rationale for creating the dataset(s) clearly described?

Partly

Are the datasets clearly presented in a useable and accessible format?

No

Are the protocols appropriate and is the work technically sound?

Partly

Reviewer Expertise:

Social responsibility, transparency, ethical codes

I confirm that I have read this submission and believe that I have an appropriate level of expertise to confirm that it is of an acceptable scientific standard, however I have significant reservations, as outlined above.

# Response

**Published:** 2023-05-05

**Authors:** Eric Jensen

**Affiliations:** Institute for Methods Innovation, Ireland

Thanks very much for your helpful and constructive feedback! I have submitted an updated version of this article. Further details about this data have also been added to the Open Access Repository: https://zenodo.org/record/6914524.

# Reviewer report: Reviewer response for version 1

**DOI:** 10.21956/openreseurope.15456.r28278

**Published:** 2022-01-13

**Authors:** Ross S. Bailie

**Affiliations:** 1 University Centre for Rural Health, University of Sydney, Lismore, NSW, Australia

This data note introduces a survey of five 'research performing organisations' that are involved in a project to embed sustainable responsible research and innovation practices. The paper is generally well-written. There are few points that should be addressed before the data note can be approved:

1. The links to the Researcher Survey and the Stakeholder Survey data show documents that appear to be identical with both being labelled 'Researcher Survey'. This needs to be corrected.
2. In the Materials and Methods section the subheadings should be refined/corrected - the text under the subheading 'Research Instrument Design'  refers to sampling of respondents and collection of the data, while info on the instrument design appears under the previous subheading. This could be fixed by removing the two level 1 subheadings and including a new subheading on 'Data Collection'.
3. The data Note could be improved by including information on inclusion/exclusion criteria for the sampling process.
4. The note could also be improved by clarification of the reference to 'department or institution wide requests for information' (in the paragraph labelled 'Sampling').
5. The Plain Language Summary refers to 'a pilot effort to implement these surveys', but there is no reference to this being a pilot in other sections of the note. It should be made clear if this was or was not a pilot study, and if so what were the aims and objectives of the pilot study.
6. It would be useful to include some commentary on lessons learned from this initial 'baseline' survey, what refinements have been or should be made to the survey methods and tools, and the extent to which the survey design and survey implementation are considered fit-for-purpose.
7. I suggest the title of the Data Note should be refined to more closely reflect the aim/purpose of the Data Note.

Are sufficient details of methods and materials provided to allow replication by others?

Partly

Is the rationale for creating the dataset(s) clearly described?

Yes

Are the datasets clearly presented in a useable and accessible format?

No

Are the protocols appropriate and is the work technically sound?

Yes

Reviewer Expertise:

Health services and systems research

I confirm that I have read this submission and believe that I have an appropriate level of expertise to confirm that it is of an acceptable scientific standard, however I have significant reservations, as outlined above.
